# Lectures on Materia Medica in the University of Buffalo

**Published:** 1858-01

**Authors:** 


*Lectures on Materia Medica in the University of Buffalo.*—Circumstances rendering it inconvenient for Prof. C. A. Lee to give the course on Materia Medica, in the University of Buffalo, during the present session, Dr. Theophilus W. Mack, of St. Catherines, C. W., was appointed by the medical faculty to officiate in his stead. Dr. Mack has given much attention to the study of chemistry and materia medica; his proficiency in these branches, together with his large experience and scholarly attainments, render him admirably fitted to discharge with ability and success the duties of a public teacher. His lectures have been received by the class with marked satisfaction.

*

---

*Dr. Reynold's Truss.*—We call the attention of our readers to the advertisement of Dr. Reynold's Truss in this number. Like all really valuable inventions it is simple in its construction, consisting of a simple band without the iron spring which has been heretofore considered a necessary evil In the band is concealed a short elastic spring, only two inches in length acting on the point of rupture by means of a block so governed that its pressure is upward and backward. The band is held in position by two perineal straps. It is the lightest truss we have seen and less liable to chafe and irritate.

---

*Jordan's Muscle.*—We see by the pages of the Virginia Medical Journal, that our poet professor, Dr. Homes, had presented a description of this muscle to the Boston Society for Medical Improvement, before it appeared in the Virginia Journal. Dr. Jordan is certainly entitled to credit for calling the attention of the profession to this anomaly, not knowing that it had been done before him; but in the article quoted from the Boston Journal, Dr. Holmes refers to Meckel, Thiele, Vesalius and John Bell, as having mentioned its existence. F.

